# An Effective Method to Detect Volatile Intermediates Generated in the Bioconversion of Coal to Methane by Gas Chromatography-Mass Spectrometry after *In-Situ* Extraction Using Headspace Solid-Phase Micro-Extraction under Strict Anaerobic Conditions

**DOI:** 10.1371/journal.pone.0163949

**Published:** 2016-10-03

**Authors:** Jianmin Liu, Baoyu Wang, Chao Tai, Li Wu, Han Zhao, Jiadong Guan, Linyong Chen

**Affiliations:** 1 Institute of Resources and Environment, Henan Polytechnic University, Jiaozuo, China; 2 National Key Laboratory of Coal and Coal-bed Methane Simultaneous Extraction, Jincheng, China; National University of Ireland - Galway, IRELAND

## Abstract

Bioconversion of coal to methane has gained increased attention in recent decades because of its economic and environmental advantages. However, the mechanism of this process is difficult to study in depth, partly because of difficulties associated with the analysis of intermediates generated in coal bioconversion. In this investigation, we report on an effective method to analyze volatile intermediates generated in the bioconversion of coal under strict anaerobic conditions. We conduct *in-situ* extraction of intermediates using headspace solid-phase micro-extraction followed by detection by gas chromatography-mass spectrometry. Bioconversion simulation equipment was modified and combined with a solid-phase micro-extraction device. *In-situ* extraction could be achieved by using the combined units, to avoid a breakdown in anaerobic conditions and to maintain the experiment continuity. More than 30 intermediates were identified qualitatively in the conversion process, and the variation in trends of some typical intermediates has been discussed. Volatile organic acids (C2–C7) were chosen for a quantitative study of the intermediates because of their importance during coal bioconversion to methane. Fiber coating, extraction time, and solution acidity were optimized in the solid-phase micro-extraction procedure. The pressure was enhanced during the bioconversion process to investigate the influence of headspace pressure on analyte extraction. The detection limits of the method ranged from 0.0006 to 0.02 mmol/L for the volatile organic acids and the relative standard deviations were between 4.6% and 11.5%. The volatile organic acids (C2–C7) generated in the bioconversion process were 0.01–1.15 mmol/L with a recovery range from 80% to 105%. The developed method is useful for further in-depth research on the bioconversion of coal to methane.

## Introduction

Coal is an important and widely distributed energy resource, and it accounts for ~30% of the global total energy consumption [1]. However, it also causes many environmental problems, such as greenhouse gas emissions, mercury pollution, and particulate pollution [2]. The conversion of coal to gas or liquid forms to reduce environmental problems has been a sustained interesting research area [3,4]. Compared with traditional thermochemical methods, bioconversion of coal to methane gas as a fuel source is believed to be advantageous because of its lower cost and reduced pollution [5–7]. Various studies have attempted to clarify the conversion process from microbiological [8], enzymatic [9], and chemical [10] perspectives. However, the conversion process is far from well understood, partly because of difficulties in the analysis of intermediates generated during coal bioconversion.

Although methane is a simple end product of coal bioconversion, the entire conversion process is complicated. In the process, complex macromolecules in coal are degraded stepwise into smaller molecules by complex anaerobic communities until methane is generated [11, 12]. Various metabolic pathways have been found to be involved in the bioconversion of coal to methane, including extracellular enzymatic depolymerization, fumarate, hydroxylation, carboxylation, methylation, and methanogenesis [13–16]. A large number of intermediates are generated and transformed in different stages, which makes a study of the mechanism very difficult [17]. Field metabolomics has been developed to elucidate the biological pathways of coal-derived methane [18], in which water produced from a well is collected, acidified, solvent extracted, and analyzed by gas chromatography-mass spectrometry (GC-MS). Using this method, benzene, toluene, ethylbenzene, and xylene compounds; polycyclic aromatic hydrocarbons; phenols; aromatic acids; alicyclic; alkane; and alkene hydrocarbons have been found in several field investigations [19–22]. These identified compounds have been very helpful for elucidating biological pathways involved in the bioconversion of coal to methane. However, field metabolomic results are static, and cannot reflect variations in trends of intermediates during bioconversion to provide a more detailed description of the bioconversion process.

To obtain a more detailed understanding of the process, laboratory simulation studies are necessary. However, it is difficult to investigate accurately varying trends in intermediates continuously during the entire bioconversion process. Firstly, complexities of coal, nutrient medium, and degradation product structures result in a complex sample matrix, which makes sample pretreatment and purification cumbersome [10]. Secondly, bioconversion of coal to methane requires anaerobic conditions. Periodic sampling from the reaction system can destroy such anaerobic conditions. To resolve this problem, a commonly used method is that of increasing parallel anaerobic bioconversion units, terminating the reaction of any one of the parallel units at various times and analyzing the solution composition in the unit to obtain a variation in trend of the intermediates [23–25]. This approach increases the experimental workload significantly. Thirdly, liquid–liquid extraction has been used to extract intermediates generated in bioconversion [23–25]. However, the extraction process is time-consuming, consumes harmful organic solvents, and the introduction of man-made error is easy. For intermediates of low concentrations (μg/L), liquid–liquid extraction can hardly provide enrichment factors that are sufficiently large for quantitative analysis, because the initial sample volume is usually only tens of milliliters [23].

Solid-phase micro-extraction (SPME) is a well-known, non-exhaustive, and environmentally friendly sample preparation technique that combines sampling, analyte extraction, and sample introduction in a single step [26–28]. Compared with traditional enrichment methods, SPME is competitive in terms of a high pre-concentration ability, low interaction with matrix (when used in headspace-mode), and short processing time. Recent studies show that SPME can capture a variety of metabolites, such as polar, nonpolar, short-lived, and unstable metabolites in the microbial system [29–31]. Advantages of SPME make this technology promising for the extraction of intermediates generated in the bioconversion of coal to methane *in situ*. However, in most headspace SPME (HS-SPME) studies, the headspace pressure has not been considered, because the extraction is usually conducted under normal atmosphere pressure (1 atm). A recent research report indicates that reducing the headspace pressure will increase the extraction efficiency of organotins by HS-SPME [32]. However, in research of coal bioconversion to methane, the headspace pressure increases continuously because of the generation of methane and carbon dioxide.

In this work, an effective method was developed to analyze volatile intermediates generated in coal bioconversion under anaerobic conditions. This method is based on intermediates extraction *in situ* using HS-SPME and detection by GC-MS. Bioconversion simulation equipment was modified and combined with an extraction device to achieve the extraction *in situ* and to avoid a breakdown of anaerobic conditions in the reaction system to ensure continuity of the bioconversion process. To study the enhanced pressure during the bioconversion of coal to methane, the influence of headspace pressure on the extraction of the analytes was also investigated. Using the proposed method, intermediates generated in the bioconversion of coal to methane were investigated qualitatively, and volatile organic acids (C2–C7) generated in the bioconversion process were also determined quantitatively.

## Materials and Methods

### Instrumentation

The intermediate product was extracted using a Supelco SPME holder fitted with an appropriate fiber (polyacrylate, PA, 85 μm; polydimethylsiloxane, PDMS, 100 μm; polydimethylsiloxane-divinylbenzene, PDMS-DVB, 65 μm). The GC-MS system was an Agilent 7890–5975, fitted with a VF-WAXms chromatographic column (30 m × 250 μm × 0.25 μm) and the NIST98 database was used (Califonia, USA). The optimized instrumental parameters are described below. The injection port temperature was 280°C. Helium gas at 1 mL/min was used as the carrier gas. For a qualitative study of the intermediates, the GC-MS was set to scan mode, with the oven temperature programmed by holding at 60°C for 3 min and ramping to 250°C at 10°C/min and then holding for 20 min. For the quantitative study of the volatile C2–C7 organic acids, the GC-MS was set to selected ion monitoring mode, with the oven temperature programmed by ramping from 60°C to 100°C at 10°C/min, holding for 3 min, ramping to 150°C at 15°C/min, and holding for 5 min with a post run at 250°C for 5 min. To simplify the method, ion fragments (m/z) of 45, 57, 60, and 73 were selected as monitoring ions for all six acids.

**Fig 1:**
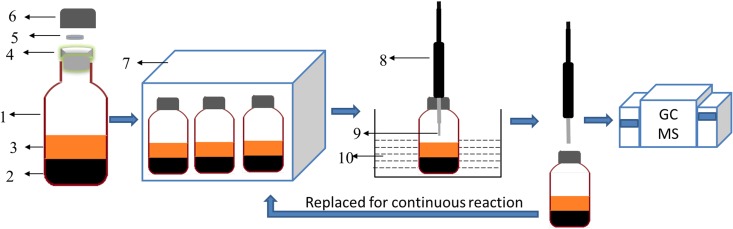
Schematic diagram of bioconversion and extraction process. 1: serum bottle, 2: coal, 3: culture solution and microflora, 4: rubber plug of the serum bottle, 5: septa silicone rubber of GC, 6: screw cap of serum bottle, 7: incubator shaker, 8: SPME holder, 9: fiber of SPME, 10: water bath.

Bioconversion simulation equipment was modified and combined with an extraction device as shown in Fig 1. A slightly modified 200 mL serum bottle was used as the anaerobic reactor and the SPME extraction container. When only the rubber plug of the serum bottle (4 in Fig 1) was used for sealing, gas leakage was possible after several extractions using SPME. The anaerobic conditions would be destroyed. So, an additional GC silicone rubber injector septa (5 in Fig 1) was added onto the rubber. After the screw cap (6 in Fig 1) had been tightened onto the serum bottle, excellent sealing could be obtained even after the SPME holder had been pierced ten times (8 in Fig 1). Serum bottles with coal and microflora were placed into the incubator shaker (7 in Fig 1) to initiate the reaction. At regular intervals, the serum bottle was removed and was placed in the water bath (10 in Fig 1) at the same temperature as the incubator shaker. Then the SPME holder (8 in Fig 1) pierced the serum bottle, and the fiber (9 in Fig 1) was exposed to the headspace in the bottle. After a certain extraction time, the intermediates were analyzed by GC-MS as described above. After extraction, the serum bottle was replaced in the incubator shaker to continue the reaction.

### Chemicals, coal samples, and microbial culture

#### Chemicals

All chemicals were at least of analytical reagent grade and were used as received. Solutions were prepared using deionized water, which was purified with a Milli-Q water treatment system (France). Acetic acid, propionic acid, butyric acid, pentanoic acid, hexanoic acid, and heptanoic acid were purchased from J&K Scientific Ltd (Beijing, China). A mixed stock solution of six organic acids was prepared, with 400 mmol/L acetic acid, 100 mmol/L propionic acid, 20 mmol/L butyric acid, 10 mmol/L pentanoic acid, 4 mmol/L hexanoic acid, and 2 mmol/L heptanoic acid. Working solutions were prepared daily by appropriate dilution of the stock solutions with deionized water. The microflora culture solution was prepared according to a method reported elsewhere [33] (for detailed information, see S1 File).

#### Coal samples and microbial source

Brown coal samples in this experiment were collected from the Yima coalfield, which is located in the northwest of Henan Province, China. The coal field longitude and latitude were E111°45′11′′-111°51′16′′ and N34°43′16′′, respectively. The coal sample had been stored outdoors for more than six months. After cleaning with pure water, the coal sample was crushed into powder using a ball mill and a sieve. Coal powder of 85–100 mesh was used in the bioconversion experiment, whereas coal powder smaller than 100 mesh was used in the bacteria acclimatization experiment. Biogas slurry that was made from swine manure and wheat straw was used as an initial microbial source. Fresh wheat straw was collected in the field in July immediately after the wheat harvest. The wheat straw was cleaned with pure water, dried naturally, and was sterilized using an autoclave. The fresh swine manure was collected from a small pig farm immediately before the acclimatization experiment. Natural plants were the main feed source at the pig farm.

#### Acclimatization of anaerobic microflora

Chopped wheat straw (500 g), fresh swine manure (500 g), and culture medium solution (10 L) were mixed with N_2_ sparging and were sealed to ferment at 35°C for 7 days. Then, 100 mL fermentation liquor was added to 500 mL of the culture medium containing 25 g brown coal powder smaller than 100 mesh. The mixture solution was incubated under anaerobic conditions at 35°C. Every 7 days, 100 mL of the mixture was transferred into 500 mL of fresh culture medium that contained 25 g coal powder for further incubation. This process was repeated five times to obtain an anaerobe seed solution to be used in the following experiments.

### Coal bioconversion and analysis of intermediates

In an anaerobic glove box, 25 g coal powder of 85–100 mesh size was mixed with 80 mL culture medium solution in 200 mL serum bottle, and then 10 mL seed solution was added. The total solution volume was ~110 mL with a headspace volume of 100 mL. The serum bottle was sealed with a rubber plug, septa silicone rubber, and screw cap. Serum bottles with coal and microflora were placed into the incubator shaker to initiate the reaction at 35°C. At regular intervals, the serum bottle was removed, placed in a water bath at the same temperature as the incubator shaker (35°C). Then the SPME holder pierced the serum bottle, and the fiber was exposed to the headspace in the bottle. After a certain extraction time, intermediates were analyzed by GC-MS as described above. Methane generated during the bioconversion process was detected using GC equipped with a thermal conductivity detector (TCD) (S2 File). The serum bottle after extraction and gas sampling was replaced into the incubator shaker to continue the bioconversion experiment.

### Optimization of extraction conditions

The extraction conditions, such as fiber selection, extraction time, solution acidity, and headspace pressure were investigated in this study. Other conditions, such as temperature, agitation, and salinity were not taken into consideration, because they were constant in the bioconversion process and could not be changed. Optimization of the extraction conditions was performed using the standard analyte solution in the 200 mL serum bottle that contained the same culture medium solution with the same solution volume and headspace volume. Higher headspace pressures (~1.5 atm and 2.0 atm) were obtained by injecting 50 mL and 100 mL N_2_ into a sealed serum bottle using a gastight syringe. A headspace pressure higher than 2 atm was not investigated because of the broken serum bottle.

## Results and Discussion

### Qualitative analysis of intermediates

**Fig 2:**
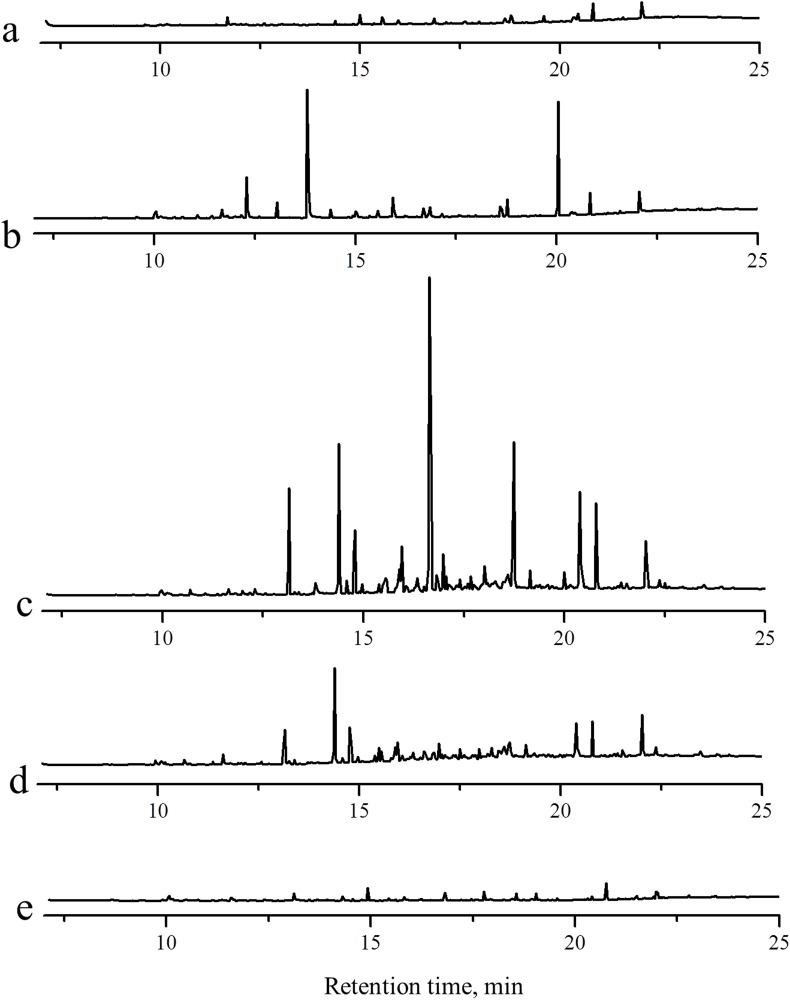
Chromatograms of the intermediates generated in coal bioconversion. a: 0 d, b: 7 d, c: 14 d, d: 21 d, e: 49 d.

Intermediates generated in the coal bioconversion were detected by GC-MS after extraction *in situ* by HS-SPME using PA fiber for 30 min, followed by preliminary qualitative analysis using the NIST98 database. Fig 2 shows chromatograms of the samples on days 0, 7, 14, 21, and 49 on the same y-axis scale. The solution composition changed substantially during the coal bioconversion process. The largest number of intermediates with a relatively high peak occurred on day 14 (Fig 2). With an initial integrator threshold of 18, more than 40 compounds could be detected on day 14. Thirty-two compound with a matching factor greater than 80% are given in Table 1. Compounds that contain benzene rings, such as substituted phenol, naphthalene, substituted naphthalenes, substituted biphenyl, fluorine, and indoles were the main intermediates on day 14. Differences in the compounds generated in the bioconversion in presence and absence of coal on the seventh and fourteenth days were also investigated.There was a significant difference between the intermediates in presence or absence of coal. In general, a greater and higher concentration of intermediates existed in the presence of coal (S1 Fig).

**Table 1:** Intermediates on the fourteenth day identified by GC-MS based on NIST98.

	Retention time, min	Compound	Peak area, %	Matching factor
1	9.662	Acetic acid	0.233	90%
2	9.965	2-Ethyl-1-hexanol	0.557	83%
3	10.684	Propanoic acid	0.523	95%
4	11.062	Propanoic acid, 2-methyl-	0.278	87%
5	11.369	Hexadecane	0.229	98%
6	11.643	Ethanol, 2-(2-ethoxyethoxy)-	0.618	86%
7	11.986	Benzene, pentamethy-	0.401	96%
8	12.364	Butanoic acid, 2-methyl-	0.400	84%
9	13.151	Naphthalene	4.703	91%
10	13.288	Cyclooctane	0.227	91%
11	13.390	Hexadecane, 2,6,10,14-tetramethyl-	0.267	91%
12	13.733	Heneicosane	0.283	90%
13	13.803	Pentanoic acid, 4-methyl	1.464	90%
14	14.384	Naphthalene, 2-methyl-	7.623	96%
15	14.795	Naphthalene, 1-methyl-	4.744	96%
16	14.996	Butylated Hydroxytoluene	0.533	98%
17	15.377	Naphthalene, 1-ethyl-	0.553	93%
18	15.960	Naphthalene, 1,3-dimethyl-	4.085	97%
19	16.063	Diphenylmethane	0.517	96%
20	16.337	Naphthalene, 1,4-dimethyl	1.601	97%
21	16.508	Naphthalene, 1,6,7-dimethyl-	1.354	94%
22	16.679	Phenol, 4-methyl-	20.203	96%
23	16.851	1,1’-Biphenyl, 4-methyl	1.521	96%
24	16.988	1,1’-Biphenyl, 2,4-dimethyl	2.525	95%
25	17.399	Naphthalene, 1,4,6-trimethyl-	1.473	96%
26	17.604	Naphthalene, 1,4,5-trimethyl-	8.005	95%
27	18.735	Dimethyl phthalate	8.347	94%
28	19.146	Fluorene	2.218	96%
29	20.002	Indole	2.218	94%
30	20.379	1 H-Indole, 3-methyl	6.524	96%
31	23.462	Phenanthrene, 2-methyl-	0.678	90%
32	25.073	Phenanthrene, 2,5-dimethyl-	0.543	93%

### Variation of typical intermediates and methane generation

**Fig 3:**
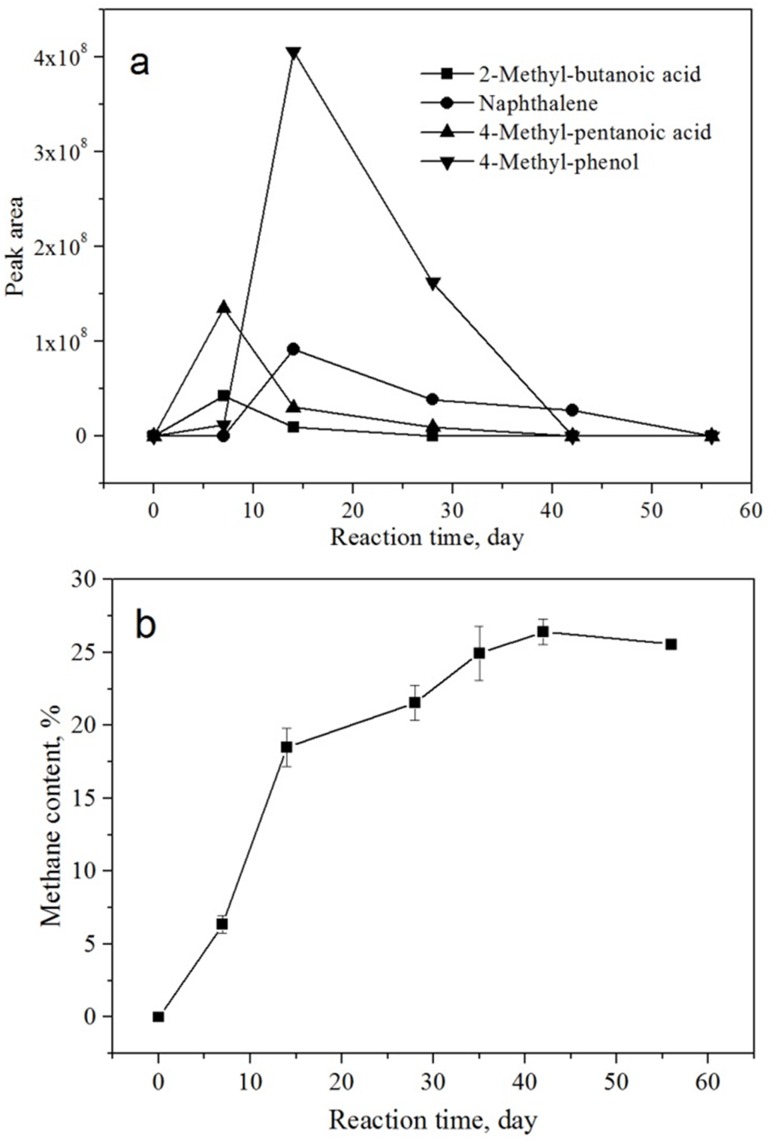
Relative changes of typical intermediates (a) and methane generation (b) over reaction time during coal bioconversion.

Of the intermediates, 2-methyl-butanoic acid, 4-methyl-pentanoic acid, naphthalene, and 4-methyl-phenol were chosen to investigate the relative content variation during the coal bioconversion process according to a change in peak area. The results are shown in Fig 3a. Different kinds of intermediates formed in the reaction solution in different periods. During the first 7 days, low molecular weight organic acids were the dominant intermediates; whereas in the later 7 days, compounds containing benzene rings were the primary products. After 42 days, all intermediates almost disappeared from solution. Using liquid–liquid extraction after GC-MS detection, similar intermediates [1, 23–25] and their variation [1] have also been found in other research. The generation of methane during the bioversion is shown in Fig 3b. Methane generation can divided into three stages (0–14 d, 14–42 d; and 42–56 d). Changes in methane generation rates agreed well with the variation in trend of the typical intermediates. The results above indicate that different kinds of intermediates may play a dominant role in the different coal bioconversion stages.

### GC-MS analysis of C2–C7 organic acid

Low molecular weight organic acids, especially acetic acid, are believed to play an important role in coal bioconversion, through a pathway known as acetoclastic methanogenesis [34, 35]. The measurement of low molecular weight organic acids is helpful to understand the coal bioconversion process. Based on the qualitative results of the intermediates above, a method to determine volatile C2–C7 organic acids contents quantitatively was developed in the following study.

Six low molecular weight organic acids were separated using the VF-WAXms chromatographic column (30 m × 250 μm × 0.25 μm), under the optimized separation condition described in the experimental section (S2 Fig). Large differences existed in the sensitivity of the method for the six organic acids. The method sensitivity increased significantly with increasing carbon number. This difference was also observed in previous research, and was proven to occur mainly because of the adsorption efficiency of different organic acids onto the fiber [36].

#### Fiber coating selection

**Fig 4:**
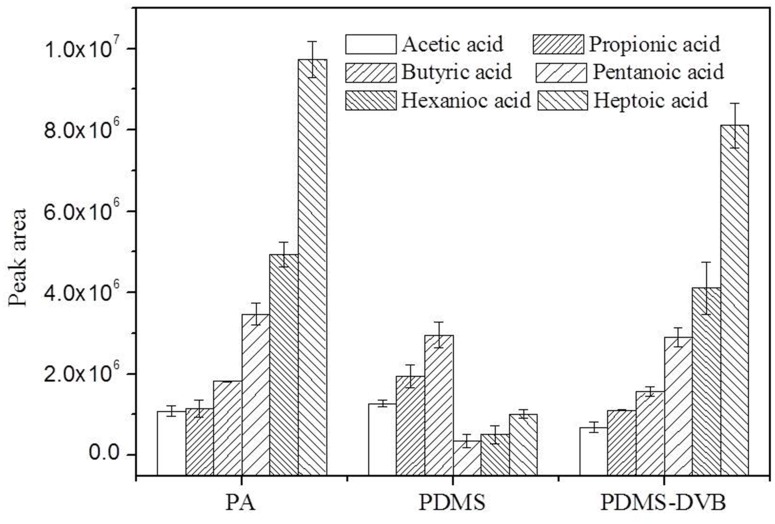
Comparison of extraction efficiency of different SPME fibers. Extraction time: 20 min, pH: 7.4, headspace pressure: 1 atm. Acetic acid (0.8 mmol/L), propionic acid (0.2 mmol/L), butyric acid (0.04 mmol/L), pentanoic acid (0.02 mmol/L), hexanoic acid (0.008 mmol/L), and heptanoic acid (0.004 mmol/L).

PDMS/DVB/CAR fiber has been found to be most efficient for the extraction of low molecular weight organic acids in a previous study [37]. However, it was difficult to reach adsorption and desorption equilibrium even after 1.5 h extraction, which was inconvenient for high-throughput analysis. Therefore, PA, PDMS, and PDMS/DVB fibers, which are often used in organic acid extraction, were selected to investigate the extraction efficiency of different fibers for the six organic acids. The results are shown in Fig 4. PDMS fiber had a higher extraction efficiency for C2–C4 organic acids than the other two fibers, but was unsuitable for the extraction of organic acids with greater carbon number. Compared with PDMS fiber, PDMS-DVB had a higher extraction efficiency for C5-C7 organic acids, but a lower extraction efficiency for C2–C4 organic acids. PA fiber had an acceptable extraction efficiency for all six organic acids. Therefore, the PA fiber was chosen for further optimization of the analytical procedure.

#### Extraction time

**Fig 5:**
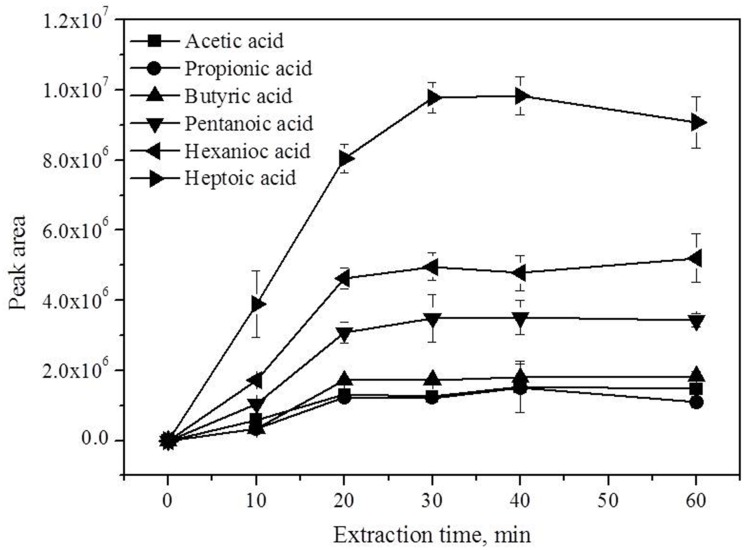
Influence of extraction time on the extraction efficiency. Fiber: PA, pH: 7.4, headspace pressure: 1 atm. Acetic acid (0.8 mmol/L), propionic acid (0.2 mmol/L), butyric acid (0.04 mmol/L), pentanoic acid (0.02 mmol/L), hexanoic acid (0.008 mmol/L), and heptanoic acid (0.004 mmol/L).

The extraction time may influence the adsorption efficiency of the organic acids onto the fiber. The influence of extraction time on extraction efficiency was investigated (Fig 5). All six organic acids, except heptanoic acid could nearly reach adsorption and desorption equilibrium in 20 min. The rapid adsorption and desorption equilibrium of organic acids onto PA fiber was also found in a previous study [36]. Considering that chromatographic separation can be completed within 20 min, the extraction time was set to 20 min to minimize the analysis time.

#### Solution acidity

Although the culture medium used in the coal bioconversion process was a buffer solution (pH 7.4), the reaction solution pH could decrease to ~4–5 under extreme conditions if the organic acids generated during the process could not be consumed in time. The influence of solution acidity on the extraction efficiency was investigated (S3 Fig). The solution acidity has very little influence on the extraction efficiency in the pH range from 4 to 7.5. The influence of solution acidity on the SPME extraction efficiency of low molecular weight organic acids was also studied in a previous report, which found that only under very low pH (1.5) and high salinity conditions, could the solution acidity influence the SPME extraction significantly [38].

#### Headspace pressure

In most HS-SPME studies, the headspace pressure was not considered, because the extraction was usually conducted under normal atmospheric pressure (1 atm). In our experiment, the headspace pressure usually increases with anaerobic reaction time (usually with a maximum final pressure of ~2.0 atm), because of the generation of methane and carbon dioxide. Therefore, the influence of headspace pressure on the extraction efficiency was investigated (S4 Fig). The headspace pressure had little influence on the extraction efficiency of all six organic acids.

Our results on the influence of headspace pressure were not consistent with the results from a previous study, in which SPME was applied in the extraction of organotin [32]. The extraction efficiency of organotin decreased when the headspace pressure increased from 0.04 to 1 bar with a short fixed extraction time. The difference in impact of the headspace pressure on the extraction efficiency between low molecular weight organic acids and organotins was possible because of the difference in volatility of these compounds. The saturated vapor pressure and response ratio under high and low pressures for the low molecular weight organic acids and organotins are listed in Table 2.

**Table 2:** Vapor pressure and response ratio between low pressure and high pressure of organotins and organic acids.

Compound^a^	VP^b^, Pa	Lp/Hp^c^	Compounds	VP^d^, Pa	Lp/Hp^e^
MBTEt_3_	22.9	1.44	Acetic acid	1520	~1
DBTEt_2_	2.42	1.50	Propionic acid	1330	~1
TBTEt	0.256	2.00	Butyric acid	100	~1
MPhTEt_3_	0.411	2.41	Pentanoic acid	19	~1
DPhTEt_2_	0.0247	5.69	Hexanoic acid	2.51	~1
TPhTEt	0.0002	8.73	Heptanoic acid	0.59	~1

^a:^ MBT, Monobutyltin; DBT; Dibutyltin; TBT; Tributyltin; MPhT, Monophenyltin; DPhT, Diphenyltin; TPhT, Triphenyltin; Et; Ethyl-.

^b:^ Vapor pressure, obtained from ref 32

^c:^ Lp/Hp, response ratio of low pressure and high pressure, data in ref. 32

^d:^ Vapor pressure obtained from NIST (http://srdata.nist.gov/gateway/)

^e:^ This study.

The organic acid volatility was significantly higher than that of the organotin compounds. It is well known that the vapor pressure changes little with external pressure for a given temperature. So, the influence of external pressure on the extraction efficiency occurs mainly because of the migration speed of the analytes from the liquid to the gas phase, but the equilibrium position will not change. For compounds with a low vapor pressure, the low external pressure will enhance analyte migration from the liquid to gas phase significantly [32]; whereas for compounds with a high vapor pressure, the influence of external pressure is small. This may explain the difference between the impact of external pressure on the organotins and organic acids extraction.

### Linearity, precision, and limits of detection

Under optimized conditions (PA fiber; extraction time, 20 min; extraction temperature, 35°C; extraction pressure, 1 atm), six low molecular weight organic acids were analyzed with different concentration ranges. The calibration curve was plotted by taking the peak area against the analyte concentration (mmol/L). The regression equation, regression coefficient, lower detection limit (LOD), lower detection quantity (LOQ), and relative standard deviation of six organic acids are shown in Table 3. The reproducibility of the method was determined by performing an extraction of the standard solution five times with the concentration given in the figure caption. The relative standard deviations ranged from 4.6 to 11.5%. The lower detection limit calculated based on three times the standard deviation for five results of the blank divided by the slope of the calibration curve ranged from 0.0006 to 0.02 mmol/L for the different organic acids.

**Table 3:** Linearity, precision and limits of detection.

Compound	Linear regression equation, mmol/L	R^2^	LOQ, mmol/L	LOD, mmol/L	RSD,% ^a^
Acetic acid	y = 3.51×10^6^x + 1.16×10^5^	0.9946	0.08	0.02	7.3
Propionic acid	y = 1.43×10^7^x + 4.68×10^4^	0.9784	0.05	0.01	5.5
Butyric acid	y = 1.63×10^8^x + 4.01×10^6^	0.9847	0.01	0.004	4.6
Pentanoic acid	y = 1.35×10^9^x + 8.09×10^5^	0.9868	0.005	0.002	7.8
Hexanoic acid	y = 3.90×10^9^x + 2.01×10^6^	0.9764	0.004	0.001	11.5
Heptanoic acid	y = 1.24×10^10^x + 8.02×10^6^	0.9631	0.002	0.0006	9.8

^a:^ Results of 0.5 mL mixed stock solution diluted into 100 mL, n = 3

### Analysis of real samples

**Fig 6:**
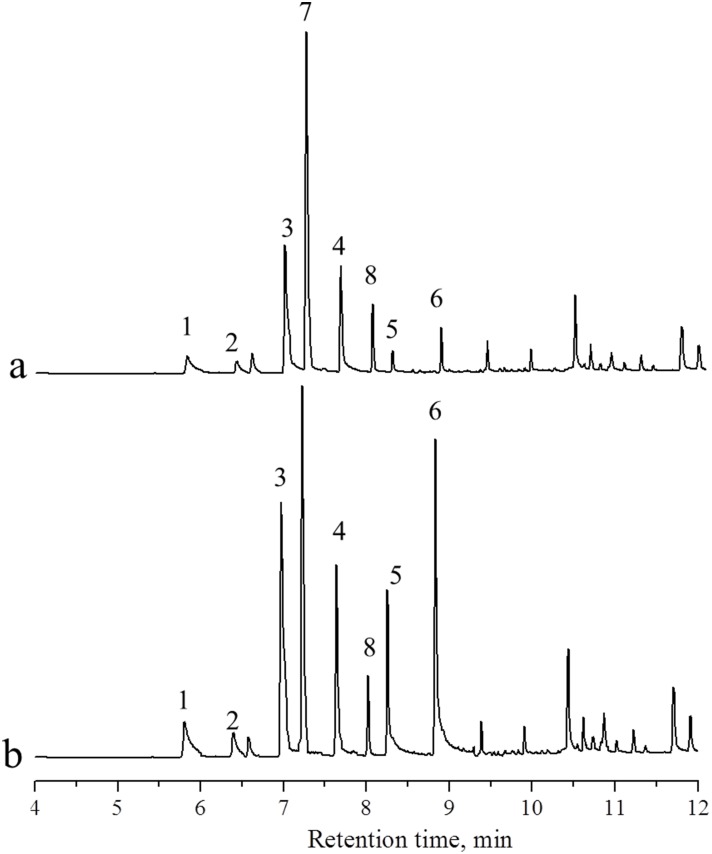
Chromatograms of the sample on day 7 (a) and the corresponding sample after standard adding (b). 1: acetic acid, 2: propionic acid, 3: butyric acid, 4: pentanoic acid, 5: hexanoic acid, 6: heptanoic acid, 7, 8: unknown

The developed procedure was applied to the *in-situ* analysis of low molecular weight organic acids generated during the coal bioconversion. A chromatogram of the sample on day 7 and the corresponding sample after standard addition is shown in Fig 6. All six low molecular weight organic acids could be detected in the sample (peaks 1 to 6). Two other unknown compounds (peaks 7 to 8) exist in the interval of peaks 1 to 6, which did not affect the quantification of peaks 1 to 6. The quantitative results of the sample and recoveries are listed in Table 4. Hexanoic and heptanoic acids were detected in the sample, but the concentrations were lower than the quantitative limit. The concentrations of acetic acid, propionic acid, butyric acid, and pentanoic acid ranged from 0.01 to 1.15 mmol/L. The recovery of the six organic acids ranged from 80% to 105%.

**Table 4:** Sample analysis and recovery.

Compound	Concentration mmol/L	Added^a^ mmol/L	Founded mmol/L	Recovery, %
Acetic acid	1.15	0.80	1.93	97.50
Propionic acid	0.12	0.20	0.28	80.00
Butyric acid	0.06	0.04	0.097	92.50
Pentanoic acid	0.01	0.02	0.028	90.00
Hexanoic acid	<0.004	0.008	0.0084	105.00
Heptanoic acid	<0.002	0.004	0.0037	92.50

^a:^ 0.2 mL mixed stock solution added

## Conclusion

An effective method to analyze volatile intermediates generated in coal bioconversion under anaerobic conditions was developed, based on intermediate *in-situ* extraction using HS-SPME and detection by GC-MS. The developed method combined bioconversion simulation equipment and an SPME extraction device to achieve *in-situ* extraction, avoid breakdown of anaerobic conditions, and maintain the continuity of the bioconversion experiment. By using the proposed method, more than 30 intermediates were identified qualitatively in the bioconversion process. Variations in typical intermediates indicated that different kinds of intermediates may play a dominant role in the different coal bioconversion stages. Volatile organic acids (C2–C7) generated in the bioconversion process were 0.01 to 1.15 mmol/L with a recovery range from 80% to 105%. The enhanced pressure during the bioconversion process had little influence on the determination of intermediates. The developed method is helpful for further in-depth research into the bioconversion of coal to methane.

## Supporting Information

S1 File Preparation of culture medium solution. (DOC)

S2 File Methane determination. (DOC)

S1 Fig Comparison of intermediates in the presence or absence of coal (a: 7 d, b: 14 d). (TIF)

S2 Fig Chromatogram of C2~C7 organic acids standard. 1: acetic acid, 4 mmol L^-1^; 2: propionic acid, 1 mmol L^-1^; 3: butyric acid, 0.2 mmol L^-1^, 4: pentanoic acid, 0.1 mmol L^-1^, 5: hexanoic acid, 0.04 mmol L^-1^, and 6: heptanoic acid, 0.02 mmol L^-1^.
(TIF)

S3 Fig Influence of solution acidity on the extraction efficiency. Fiber: PA, extraction time: 20 min, headspace pressure: 1 atm; acetic acid (0.8 mmol L^-1^), propionic acid (0.2 mmol L^-1^), butyric acid (0.04 mmol L^-1^), pentanoic acid (0.02 mmol L^-1^), hexanoic acid (0.008 mmol L^-1^), heptanoic acid (0.004 mmol L^-1^).
(TIF)

S4 Fig Influence of headspace pressure on extraction efficiency. Fiber: PA, extraction time: 20 min, pH: 7.4; acetic acid (0.8 mmol L^-1^), propionic acid (0.2 mmol L^-1^), butyric acid (0.04 mmol L^-1^), pentanoic acid (0.02 mmol L^-1^), hexanoic acid (0.008 mmol L^-1^), and heptanoic acid (0.004 mmol L^-1^).
(TIF)
