# Supplementary material for: An Effective Method to Detect Volatile Intermediates Generated in the Bioconversion of Coal to Methane by Gas Chromatography-Mass Spectrometry after In-Situ Extraction Using Headspace Solid-Phase Micro-Extraction under Strict Anaerobic Conditions
Source: PLoS One. 2016 Oct 3;11(10):e0163949. doi: 10.1371/journal.pone.0163949 (PMC5047463; doi:10.1371/journal.pone.0163949)
Supplement: S1 File — (DOC) [file pone.0163949.s001.doc]

**S1 File. Preparation of culture medium solution**

The culture medium solution for the microflora was prepared by mixing 1 L basal medium with 100 μL trace element solution and 10 μL vitamin (Sinopharm, Beijing, China) solution. The basal medium contained (L^-1^) 2 g yeast extract, 0.5 g ascorbic acid, 0.5 g EDTA, 1.6 g K_2_HPO_4_, 1.5 g KH_2_PO_4_, 0.4 g NH_4_Cl, and 0.4 g MgCl_2_. The trace element solution contained (L^-1^) 21 g FeSO_4_·7H_2_O, 1 g MnCl_2_·4H_2_O, 1.9 g CoCl_2_·6H_2_O, 1 g KAl(SO_4_)_2_, 0.07 g ZnCl_2_, 0.02 CuCl_2_·2H_2_O, 0.24 g NiCl_2_·2H_2_O, 0.36 g Na_2_MoO_4_·2H_2_O, and 0.06 g H_3_BO_3_. The vitamin solution contained (L^-1^) 0.5 g lipoic acid, 0.01 g vitamin B12, 1 g pyridoxine hydrochloride, 0.2 g biotin, 0.5 g nicotinic acid, 0.2 g folic acid, 0.5 g calcium pantothenate, and 0.5 g p-aminobenzoic acid. All liquid media were sparged with N_2_ at the rate of 50 mL min^-1^ for 30 min and then placed into the anaerobic glove box for one night to remove the dissolved oxygen.
