# Supplementary material for: An Effective Method to Detect Volatile Intermediates Generated in the Bioconversion of Coal to Methane by Gas Chromatography-Mass Spectrometry after In-Situ Extraction Using Headspace Solid-Phase Micro-Extraction under Strict Anaerobic Conditions
Source: PLoS One. 2016 Oct 3;11(10):e0163949. doi: 10.1371/journal.pone.0163949 (PMC5047463; doi:10.1371/journal.pone.0163949)
Supplement: S2 File — (DOC) [file pone.0163949.s002.doc]

**S2 File. Methane determination**

A US Agilent 7890 gas chromatograph fitted with a Carbonplot chromatographic column (60 m × 320 μm × 1.5 μm), TCD detector and an airtight needle for sample injection were used for the gas content determination. The injection volume was 0.5 mL. The chromatographic injection port temperature was 150°C, the column oven temperature was 25°C, and the detector temperature was 200°C.
