# Supplementary figures and images for: An Effective Method to Detect Volatile Intermediates Generated in the Bioconversion of Coal to Methane by Gas Chromatography-Mass Spectrometry after In-Situ Extraction Using Headspace Solid-Phase Micro-Extraction under Strict Anaerobic Conditions
Source: PLoS One. 2016 Oct 3;11(10):e0163949. doi: 10.1371/journal.pone.0163949 (PMC5047463; doi:10.1371/journal.pone.0163949)

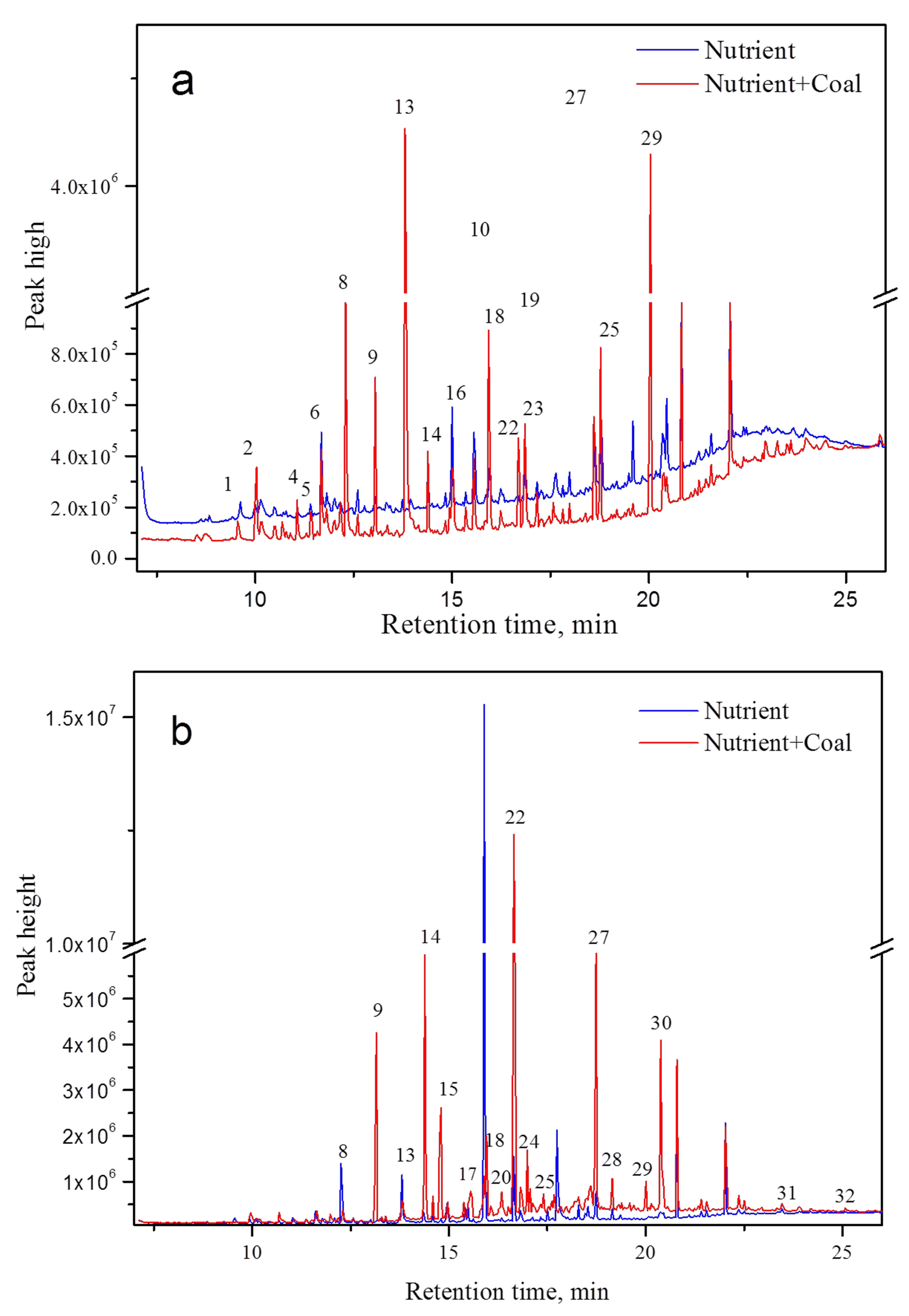

Supplement: S1 Fig — (TIF) [file pone.0163949.s003.tif]

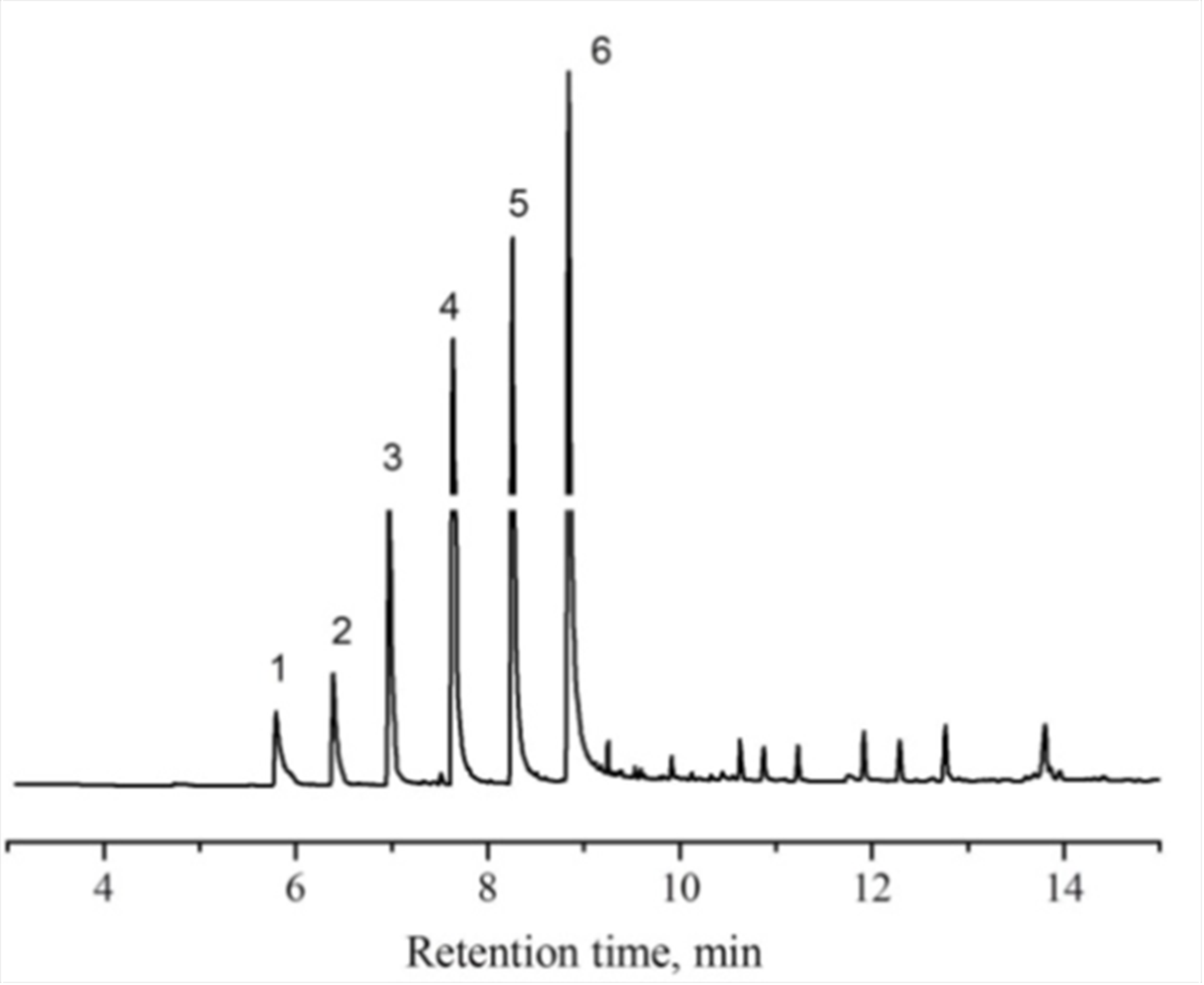

Supplement: S2 Fig — 1: acetic acid, 4 mmol L-1; 2: propionic acid, 1 mmol L-1; 3: butyric acid, 0.2 mmol L-1, 4: pentanoic acid, 0.1 mmol L-1, 5: hexanoic acid, 0.04 mmol L-1, and 6: heptanoic acid, 0.02 mmol L-1. (TIF) [file pone.0163949.s004.tif]

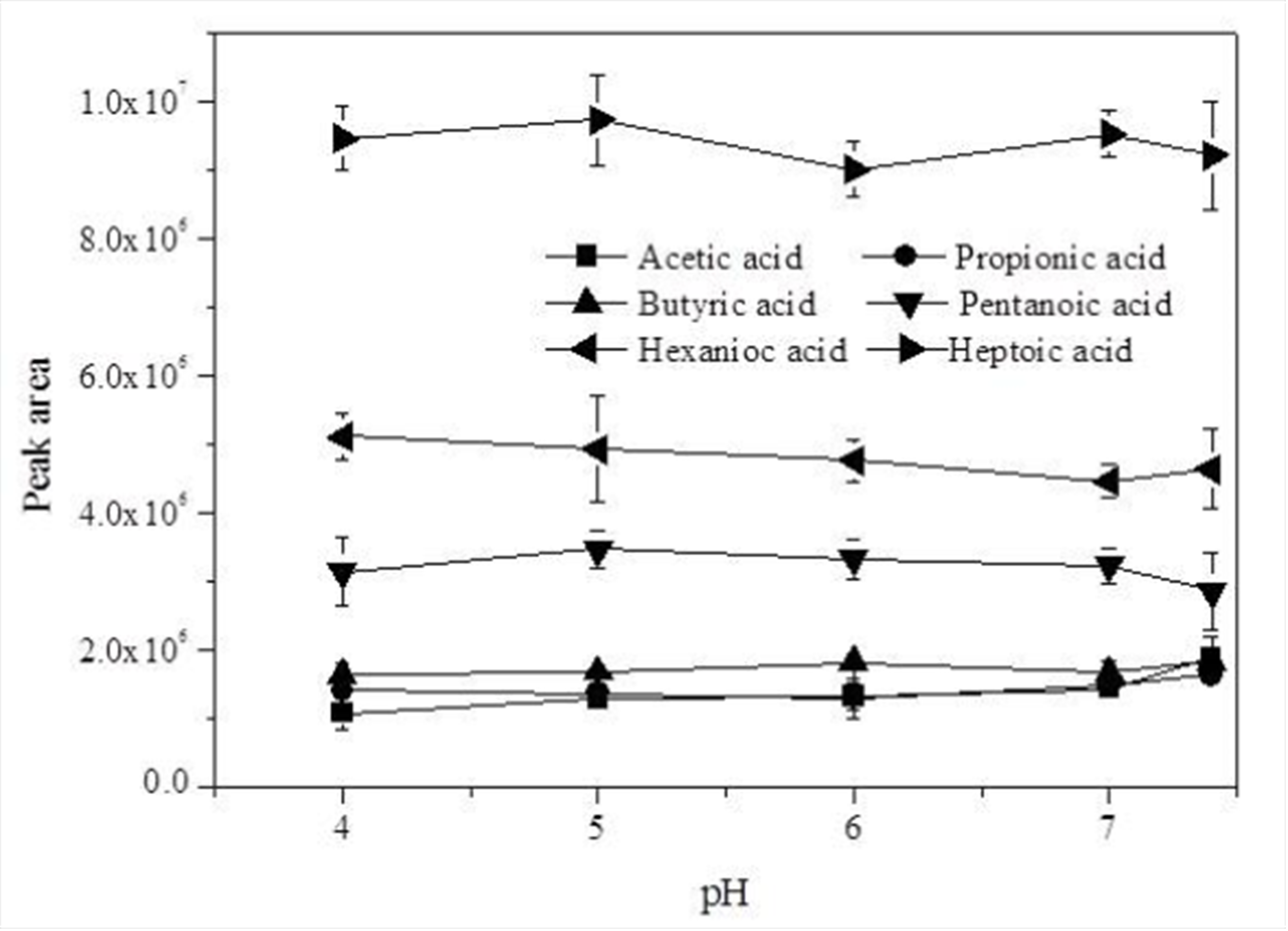

Supplement: S3 Fig — Fiber: PA, extraction time: 20 min, headspace pressure: 1 atm; acetic acid (0.8 mmol L-1), propionic acid (0.2 mmol L-1), butyric acid (0.04 mmol L-1), pentanoic acid (0.02 mmol L-1), hexanoic acid (0.008 mmol L-1), heptanoic acid (0.004 mmol L-1). (TIF) [file pone.0163949.s005.tif]

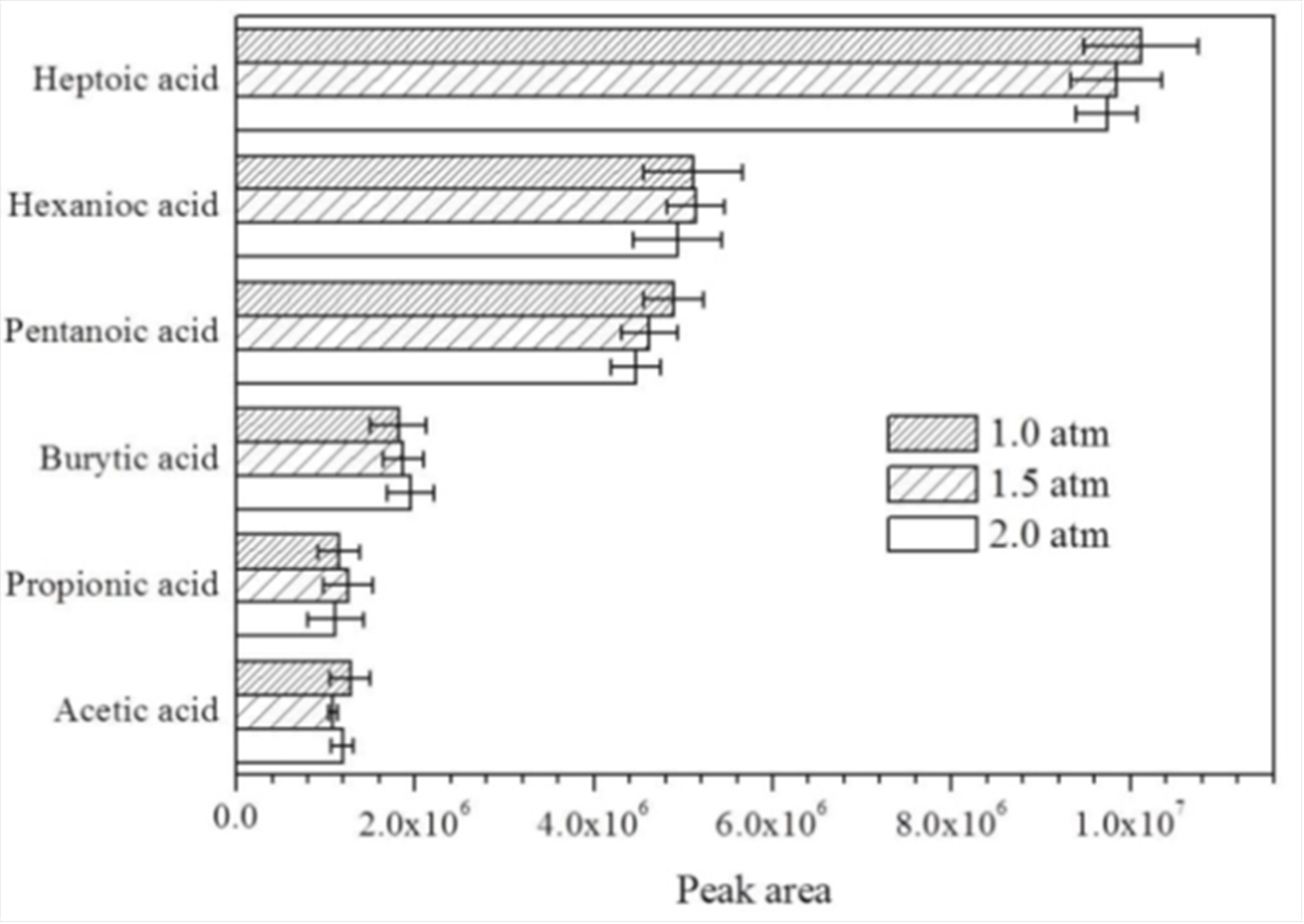

Supplement: S4 Fig — Fiber: PA, extraction time: 20 min, pH: 7.4; acetic acid (0.8 mmol L-1), propionic acid (0.2 mmol L-1), butyric acid (0.04 mmol L-1), pentanoic acid (0.02 mmol L-1), hexanoic acid (0.008 mmol L-1), and heptanoic acid (0.004 mmol L-1). (TIF) [file pone.0163949.s006.tif]
